# Phytochemical profile and in vitro evaluation of cassava (*Manihot esculenta* Crantz) foliage as ruminant feed with/without green banana flour

**DOI:** 10.1038/s41598-024-82450-3

**Published:** 2025-01-03

**Authors:** Mohamed Rashid, Hadeer M. Aboshady, Yosra A. Soltan, Harry Archimede, Wafaa M. A. Ghoneem

**Affiliations:** 1 Regional Center for Food and Feed, Agricultural Research Center, Giza, 12619 Egypt; 2 Animal Production Department, Faculty of Agriculture, Cairo University, Giza, 12613 Egypt; 3 Animal and Fish Production Department, Faculty of Agriculture, Alexandria University, Alexandria, 21526 Egypt; 4 INRAE, ASSET, Agroécologie, Génétique et, Systèmes d’Élevage Tropicaux, Guadeloupe, 97170 France

**Keywords:** *Manihot esculenta* foliage, Green banana flour, In vitro evaluation, Phytochemical evaluation, Phenolic compounds, Analytical biochemistry, Mass spectrometry, Secondary metabolism, Animal behaviour

## Abstract

Cassava (*Manihot esculenta* Crantz) is a crucial crop in tropics and subtropics, primarily cultivated for its tuber. However, its foliage is rich in protein and can supply essential elements for ruminants. The objective of this study was to evaluate the phytochemical compounds by Gas chromatography-MS (GC-MS) and the main phenolic by High Pressure Liquid Chromatography (HPLC) present in cassava foliage, along with the fermentation pattern using a semi-automated gas production (GP) system. The in vitro evaluation was carried out for four diets formulated as follows: T_1_ (alfalfa: grass hay at ratio of 30: 70); T_2_ (alfalfa: grass hay: banana flour 30:60:10); T_3_ and T_4_ (replaced alfalfa in T_1_ and T_2_ with cassava foliage, respectively). The addition of green banana flour aimed to increase the diets’ energy. The GC-MS results indicated that cassava foliage showed a large number of valuable bioactive components, with the biflavonoid isoginkgetin representing the major component at 25.33% of total peak area percentage. The HPLC analysis declared that rutin, gallic acid, and ferulic acid were the main phenolic compounds presented in cassava foliage ethanolic extract. The accumulative gas after 24 h of incubation was significantly lower with cassava diets compared to alfalfa diets, being 119.3 versus 130.1 ml/g DM, respectively. The degradation of both organic matter and neutral detergent fiber was significantly higher with alfalfa compared to cassava diets, while there was no significant difference between alfalfa and cassava diets on final pH, ammonia concentration and protozoal count. Banana flour inclusion, regardless of the forage type, decreased the accumulative gas after 24 h of incubation with about 9% compared with no banana addition. The use of cassava foliage in ruminant diets considered a promising protein source with valuable bioactive components that could have a positive effect on animal health and production.

## Introduction

The feed gab is the biggest challenge for livestock production in developing countries^1^. Besides the negative effects of climate changes and other circumstances (i.e. coronavirus disease and Russia-Ukraine war) on feed availability and prices, by-products of the agricultural sector could play a major role in narrowing the feed gap. Cassava (*Manihot esculenta*), is a staple annual root crop grown and distributed in tropical countries^2^. The production of cassava has been increasing in the last decades, with global production reaching approximately 315 million tons in 2021 ^3^. Cassava foliage is generated in significant quantities during cassava tuber production, and this foliage is usually left in the farmlands after harvesting of cassava tuber^4^.

From a nutritional standpoint, cassava foliage can serve as an animal feed due to its high protein content, ranging from 17.7 to 38.1% on dry matter basis^5^, as well as significant levels of gross energy and mineral elements^6^. Like other plants of the genus *Manihot*, cassava foliage may contain cyanogenic glycosides (i.e. linamarin and lotaustralin), which limit the consumption of the fresh foliage. However, propitiously, the cyanide produced in the foliage is a volatile compound and quickly disappears after crushing. Thus, drying foliage has the potential to reduce the toxicity and increase forage support for livestock^7^. The partial replacement of 50% of soybean meal crude protein by cassava foliage has been tested in growing goats with a similar feed efficiency^8^. Furthermore, a study by^9^ highlighted the potential of tannin-rich plants, including *Leucaena leucocephala*, *Glyricidia* sepium and *Manihot esculenta*, to reduce enteric methane (CH_4_) emissions in sheep. Additionally^10^, noted that cassava foliage mitigates CH_4_ production from sheep with no detrimental effects on other rumen fermentation parameters. The results of a recent study conducted by^11^ illustrated that the cassava foliage has a good concentration of phytochemicals with high antioxidant activity, however, a clear identification of the bioactive compounds present in cassava foliage are not reported.

Cull banana fruits, also known as non-marketable fruits, represent a significant by-product of the agriculture sector in tropical and subtropical countries. The Latin America and Caribbean countries are the world’s most important exporters of bananas, with 30–40% of banana fruits production being rejected due to failing to meet quality standards^12^. Rejected banana is classified as a starch source with a slow rate of degradation^13^. Green banana fruits, containing 70% of dry matter in non-structural carbohydrates, have a high energy content^14^. This characteristic makes discarded banana fruits a potentially valuable source of energy in animal rations^15^. The presence of banana fruits, combined with a high soluble nitrogen source such as legumes, enhances rumen microbial nitrogen efficiency by providing a highly available source of energy^14^. The effects of utilizing overripe pulp, green peel extract and powder of banana fruit in Holstein dairy calves were investigated in the study by^16^. The results showed beneficial effects on the hematological, immunological, health and average daily weight gain of calves fed banana fruits compared with the control group. These advantageous effects have been attributed to the bioactive component of banana fruits, such as flavonoids, polyphenols, and phenolic compounds^16^. The aim of the present work was to identify the bioactive compounds of cassava foliage using GC-MS and HLPC. Additionally, we aimed to compare the use of cassava foliage versus alfalfa in ruminant ration with or without green banana flour by in vitro gas production technique.

## Materials and methods

This work has been done in-cooperation between Agroecology, Genetic and Tropical Livestock Farming System (ASSET), INRAE, Petit-Bourg, Guadeloupe and the Regional Center for Food and Feed (RCFF), Agricultural Research Center (ARC), Giza, Egypt. The in vitro assay was carried out at the Advanced Laboratory of Animal Nutrition, Department of Animal and Fish Production, Faculty of Agriculture, Alexandria University, Egypt. All the procedures and experimental protocols described here were approved by the guidelines of the Directive 2010/63/EU of the European Parliament.

### Experimental feed ingredients

The experimental feed ingredients were collected in the mid of 2021 from Guadeloupe, French West Indies (Guadeloupe, latitude 16.16 N, longitude 61.30 W). The grass hay was based on tropical grass (*Dichanthium spp*.) came from natural grassland in Basse-Terre, west Guadeloupe, with irrigation and mineral fertilization around 100 kg of Nitrogen /ha/year with 75 days old. Cassava foliage was collected manually from cassava farmland in Basse-Terre, Guadeloupe. The foliage was wilted and dried in a shadow place for seven days with daily overturn. Rejected green banana fruits were obtained from a commercial farm in Guadeloupe then chopped and dried in the oven at 50 °C for 48 h. All feed ingredients were crumbled into small particles then milled through a 1 mm screen before using.

Four experimental diets were prepared as follows, T_1_ consist of alfalfa: grass hay at ratio of 30: 70; T_2_ consist of alfalfa: grass hay: green banana flour at ratio of 30:60:10; in T_3_ and T_4_ diets alfalfa replaced in T_1_ and T_2_ with cassava foliage, respectively. Diets were formulated to cover the NRC requirements for growing lambs in the range of 20–30 kg and the expected average daily gain (ADG) typically ranges from 100 to 250 g/day^17^.

### Chemical analysis of the experimental diets

The experimental diets were chemically analyzed according to Association of Official Agricultural Chemists^18^ for dry matter (DM) content by oven-drying to a constant weight at 60 °C^19^, ash content determined by burning feeds samples at 550 °C for 4 h, crude protein by Kjeldahl procedure (CP: *N* ×6.25), and ether extract (EE) with determined using an automated Soxtec apparatus (SoxtecTM2050, Foss). Contents of neutral detergent fiber (NDF), acid detergent fiber (ADF), and acid detergent lignin (ADL) were sequentially measured using the ANKOMDELTA Automated Fiber Analyzer with pump system (ANKOM, model DELTA, Macedon NY, USA) in a fiber filter bag 25-micron porosity F57 ANKOM).

Total phenols content (ascorbic acid equivalents) in the experimental diets were determined according to the Folin-Ciocalteureagent method^20^. Total tannins were estimated by the following procedure: 5 g of the sample was boiled in water for 30 min then centrifuged at 2,000 rpm for 20 min. one ml of the sample extract was added to 75 ml water then 5 ml of Folin-Denis reagent, 10 ml of sodium carbonate solution were added. The sample absorbance is read at 700 nm after 30 min and tannic acid was used as standard. The tannin content of the samples as % was obtained from the standard graph of tannic acid^21^. The chemical composition of the formulated experimental diets is illustrated in Table 1.

**Table 1:** Components (% DM) and chemical composition (g/kg DM) of the experimental diets.

Diet composition				
	T_1_	T_2_	T_3_	T_4_
	*Experimental diets*			
Alfalfa	30	30	-	-
Grass hay	70	60	70	60
Banana flour	-	10	-	10
Cassava foliage	-	-	30	30
	*Chemical composition (g/kg DM)*			
Organic matter	919.0	923.0	909.1	913.1
Crude protein	134.4	126.8	145.5	137.9
Ether extract	41.2	38.1	51.7	48.6
NDF	530.4	477.6	495.6	442.8
ADF	337.9	304.5	314.2	280.8
ADL	90.2	82.2	93.4	85.3
Hemicellulose	192.7	170.9	181.5	159.7
Cellulose	247.6	224.6	220.9	197.9
Lignin	56.0	52.6	58.0	54.7
Total phenols	6.6	6.0	11.9	11.3
Total tannins (%)	1.2	1.1	1.5	1.4

NDF: Neutral detergent fiber, ADF: Acid detergent fiber, ADL: Acid detergent lignin, Hemicellulose: NDF-ADF, Cellulose: ADF-ADL, Total phenols determined as eq- to Gallic acid (g)/DM (kg).

T_1_: alfalfa: grass hay at ratio of 30: 70, T_2_: alfalfa: grass hay: banana flour 30:60:10, T_3_ and T_4_ diets prepared by replacing alfalfa in T_1_ and T_2_ with cassava foliage, respectively.

### Phenolic compounds, HPLC quantitative analysis

The phenolic compounds existed in the ethanolic extract of cassava foliage were identified using High-Performance Liquid Chromatography (HPLC) (Agilent 1260 series Santa Clara, USA). The separation was carried out using the C18 column (Eclipse: 4.6 mm × 250 mm i.d., 5 μm) and the temperature was maintained at 40 °C. The mobile phase consisted of water (A) and 0.05% tri-fluoro-acetic acid in acetonitrile (B) at a flow rate of 1 ml/min. The mobile phase was programmed consecutively in a linear gradient as follows: 0 min (82% A); 0–5 min (80% A); 5–8 min (60% A); 8–12 min (60% A); 12–15 min (82% A); 15–16 min (82% A) and 16–20 min (82% A). The multi-wavelength detector was monitored at 280 nm. The injection volume was 5 µl of sample. The analytical curve was prepared by dilutions of 17 analytical HPLC grade phenolic standards ≥ 95% purity (Sigma-Aldrich^®^ Brand, Santa Louis, USA).

### Gas chromatography-mass spectrometry (GC-MS) analysis

These determinations were conducted by standard qualitative methods described by^21^ with optimization by a positive control. Gas chromatography-mass spectrometry (Agilent Technologies 7890 A) analysis the chemical composition of the most potent ethanolic extract, using a direct capillary column TG–5MS (30 m × 0.25 mm × 0.25 μm film thickness). Helium was used as a carrier gas at a constant flow rate of 1 ml/min. The electron ionization mass spectra were collected at 70 eV ionization voltages over the range of m/z 50–500 in full scan mode. The ion source temperature was set at 200 °C. The injected volume was 1 µl of sample extract. The components were identified by comparison of their mass spectra and retention times with those of authentic compounds and by computer matching with both mass spectral libraries NIST 11 and WILEY 09.

### In vitro gas production assay

The experimental diets were evaluated using the semi-automatic gas production system, which is equipped with a pressure transducer and a data logger (Pressure Press Data GN200, Sao Paulo, Brazil) according to^22^ and modified by^23^. Ruminal contents from five healthy fasted slaughtered buffalo bulls (*Bubalus bubalis*) with an average live weight of 500 ± 10 kg SE were obtained individually at the slaughterhouse of the Agricultural Experimental Station of the Faculty of Agriculture, Alexandria University, Egypt. The animals were fed a diet with a 40:60 roughage: concentrate ratio, clover hay as a roughage source, and a commercial concentrate fed mixture (152 CP g/kg DM). The ruminal contents were collected as described by^24^.

The pH of the ruminal fluid for animals was measured using a portable pH meter (GLP 21 model; CRISON INSTRUMENTS, Barcelona, Spain). The collected ruminal fluid was blended for 10 s, squeezed through 4 layers of cheesecloth, and the temperature was kept at 39 °C under CO2. The nutritive buffer incubation medium was prepared according to^25^ and used to dilute the squeezed rumen fluid with a 1:2 ratio (rumen: buffer) to prepare the buffered ruminal inoculum (BRI). In total we obtained five ruminal inocula, each ruminal inoculum was prepared from one animal. This was done to avoid the impacts of unusual rumen inocula in the in vitro assays^26^.

A dried ground (1 mm screen) sample of 500 mg for all the experimental diets were weighed (with eight repetitions/ruminal inoculum) in 120 ml volume dark glass bottles, and 45 ml of BRI was added then the bottles were shaken well. To obtain the net values of gas production (GP), blank bottles were prepared with 45 ml BRI without substrate. Also, clover (*Trifolium alexandrinum* L.) hay was used as an internal standard to detect the sensitivity changes induced by the BRI. After the addition of BRI, all bottles were immediately sealed with 20 mm butyl septum stoppers and incubated for 48 h at 39 °C in a forced-air oven (FLAC STF-N 52 Lt, Treviglio, Italy). The head space gas pressure was recorded at 4, 8, 12, and 24 h after the incubation start time to calculate the net produced gas volumes. After each gas sampling, the incubated bottles were vented, handily shaken, and returned to the incubator^27^.

### In vitro ruminal nutrient degradability, fermentation parameters, and protozoal count

All the incubation bottles were removed from the incubator after 48 h and placed directly on an ice bath to inhibit fermentation. After bottles opening, the ruminal final pH values were determined by the same portable pH meter which used before. The degraded organic matter (DOM) was determined following the method described by^28^. The residual (non-degraded) contents of each bottle were treated with a neutral detergent solution at 90 °C for 1 h. After this treatment, the residues were filtered using pre-weighed crucibles, thoroughly washed with hot distilled water to remove any remaining detergent, and then dried. Following drying, the samples were incinerated to ash. The DOM was calculated as the difference between the initial OM content and the amount of non-degraded OM remaining after 48 h of incubation. Similarly, degraded neutral detergent fiber (DNDF) was determined as the difference between the initial NDF content and the amount of non-degraded NDF.

Ruminal ammonia concentrations were determined calorimetrically by a commercial enzymatic kit (Biodignstic inc, Alexandria, Egypt). Neubauer improved bright-line counting chamber, and methyl green-formalin-saline solution was used to count protozoa microscopy as described by^29^. The short-chain fatty acids (SCFAs) were measured using a gas chromatograph (Thermo TRACE 1300, Rodano, Milan, Italy) and equipped with a capillary column (TRFFAP 30 m × 0.53 mm ID × 0.5 μm film (Thermo-part No: 260N225P) following the method of Palmquist and Conrad^30^.

#### Statistical analysis

The in vitro assay was completed in one day and the different diets parameters comparisons were analyzed with a factorial model using the PROC MIXED procedure of SAS (SAS Institute Inc. 2014. SAS^®^ OnDemand for Academics. Cary, NC)^31^, the model included the fixed effects of the forage type (alfalfa or cassava), with/without banana, and their interactions. The incubation bottle was the experimental unit. Comparisons of differences among treatments were considered significant at *p* ≤ 0.05 using Duncan’s Multiple Range test^32^.

## Results

### Chemical composition of the tested diets

The chemical composition of the tested diets used in the in vitro evaluation is provided in Table 1. The cassava diets (T_3_ and T_4_) were almost similar to alfalfa diets (T_1_ and T_2_) for chemical composition except for total phenols which was near to be double in cassava diets compared with alfalfa diets. The current results revealed that the highest OM content and the lowest values of other chemical compositions of banana flour were reflected on the chemical composition of diets contained banana flour (T_2_ and T_4_) comparing to the same diets without banana flour (T_1_ and T_3_).

### The most abundant bio-active compounds in cassava foliage using GC-MS

The most abundant active chemical components that have been identified in the ethanolic extract of cassava foliage are presented in Table 2. The qualitative analysis by GC-MS for cassava foliage was able to identify 26 compounds. Isoginkgetin recorded the highest peak area (25.33% of total peak area), following by retinal with 10.2% of total peak area %. Scutellarein tetramethyl ether, the powerful anti-inflammatory bioactive component, recorded 7.0% of the total peak area.

**Table 2:** The most abundant chemical compounds in the ethanolic extract of cassava foliage by GC-MS.

Active compound	*R*.T	Area%	M.F	M.W
Gardenin	10.26	0.78	C_21_H_22_O_9_	418.4
Hesperetin dihydrochalcone	11.949	0.63	C_16_H_16_O_6_	304.29
Citronellyl tiglate	12.753	2.9	C_15_H_26_O_2_	238.37
Hexa-hydro-farnesol	12.937	2.1	C_15_H_32_O	228.414
Epiglobulol	13.036	1.74	C_15_H_26_O	222.37
Corymbolone	13.307	2.72	C_15_H_24_O_2_	236.35
Scutellarein tetramethyl ether	13.692	7.0	C_19_H_18_O_6_	342.3
Phytol	14.484	2.12	C_20_H_40_O	296.5
5β,7βH,10α-Eudesm-11-en-1α-ol	14.734	4.69	C_15_H_26_O	222.36
Isolongifolol	14.767	5.76	C_15_H_26_O	294.5
Cyanidin-3-O-rhamnoside cation	15.198	2.28	C_21_H_21_O_10_+	433.4
Petunidin 3-glucoside cation	16.03	2.04	C_22_H_23_O_11_+	463.4
Stigmasterol	16.382	1.19	C_29_H_48_O	412.7
3-(3,4-Dimethoxyphenyl)-4-methylcoumarin	17.256	2.01	C_18_H_16_O_5_	312.32
5,7-Dihydroxy-3’,4’,5’-trimethoxyflavanone	17.49	2.49	C_18_H_16_O_7_	344.3
Isoginkgetin	18.802	25.33	C_32_H_22_O_10_	566.5
Vitamin E	18.281	2.08	C_29_H_50_O_2_	430.71
7,3’,4’,5’-Tetramethoxyflavanone	19.86	2.24	C_19_H_18_O_6_	342.3
Delphinidin 3-galactoside cation	20.246	2.19	C_21_H_21_O_12_	500.8
Quercetin 3’-methyl ether	21.39	2.86	C_16_H_12_O_7_	316.26
Retinal	21.96	10.2	C_20_H_28_O	284.436
β Carotene	22.214	2.43	C_40_H_56_	536.9
Supraene	21.558	2.48	C_30_H_50_	410.718
Geranyl isovalerate	22.514	4.75	C_15_H_26_O_2_	238.37
3’,4’,5’,5,6,7-Hexamethoxyflavone	22.936	1.9	C_21_H_22_O_8_	402.39
Vitexin	23.305	3.1	C_21_H_20_O_10_	432.4

R.T: retention time, M.F: molecular formula, M.W: molecular weight (g/mol).

### Phenolic components

**Fig. 1:**
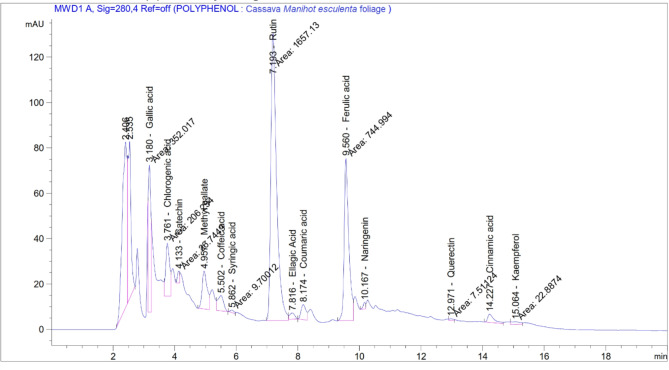
Chromatograms for the polyphenols in the ethanolic extract of cassava foliage by HPLC.

From a quantitative view, the analysis for 17 phenolic components determined in the ethanolic extract of the cassava foliage is illustrated in Fig. 1; Table 3. Cassava foliage showed a high concentration of rutin, gallic acid and ferulic acid being 814.98, 144.25 and 118.81 µg/ml of the extract, these components displayed a wide range of biological activities.

**Table 3.** Phenolic components concentration in the ethanolic extract of cassava foliage using HPLC.

Phenolic components	Retention time (min)	Molecular formula	Molecular weight (g/mol)	Concentration (µg/ml)
Gallic acid	3.04	C_7_H_6_O_5_	170.12	144.25
Chlorogenic acid	3.80	C_16_H_18_O_9_	354.31	65.88
Catechin	4.123	C_15_H_14_O_6_	290.26	12.81
Methyl gallate	5.00	C_8_H_8_O_5_	184.15	9.63
Caffeic acid	5.39	C_9_H_8_O_4_	180.16	13.80
Syringic acid	5.88	C_9_H_10_O_5_	198.17	1.92
Pyro catechol	6.05	C_6_H_6_O_2_	110.1	ND
Rutin	7.15	C_27_H_6_O_5_	610.5	814.98
Ellagic acid	7.91	C_7_H_30_O_16_	302.19	11.49
Coumaric acid	8.15	C_9_H_8_O_3_	164.16	4.95
Vanillin	8.76	C_8_H_8_O_4_	168.14	ND
Ferulic acid	9.48	C_10_H_10_O_4_	194.18	118.81
Naringenin	10.13	C_15_H_12_O_5_	272.25	2.93
Querectin	12.96	C_15_H_10_O_7_	302.2	1.90
Cinnamic acid	14.19	C_9_H_8_O_2_	148.15	1.99
Kaempferol	15.35	C_15_H_10_O_6_	286.2	4.37
Hesperetin	15.88	C_16_H_14_O_6_	302.27	ND

ND: not detected.

### In vitro gas production of tested diets

Table 4 shows the gas production (ml/g DM) after 4, 8, 12, 24 h and total accumulative after 24 h for alfalfa and cassava diets. The GP results illustrated that there was insignificant difference between experimental diets in GP at all measured times and the accumulative GP at 24 h. The incubation of cassava diets (T_3_, T_4_) significantly decrease GP compared to alfalfa diets (T_1_, T_2_) at 12, 24 h and the accumulative GP at 24 h. Cassava diets (30% of diet DM) recorded about 8.3% reduction in accumulative GP at 24 h comparing to alfalfa diets. On the other hand, the effect of banana flour addition in diets, regardless the forage type, significantly (*P* < 0.05) increased the accumulative GP at 24 h being 118.9 ml/g DM for diets without banana compared to 130.5 ml/g DM for diets with banana flour addition.

**Table 4:** In vitro gas production for alfalfa versus cassava diets.

	Gas production (ml/g DM)				
Diets	4 hours	8 hours	12 h	24 h	Accumulative 24 h
T_1_	40.2	14.5	36.3	23.9	123.8
T_2_	46.3	17.3	33.9	27.1	136.4
T_3_	45.0	13.4	28.9	19.0	114.0
T_4_	50.8	13.5	31.0	23.3	124.7
*P*-value	0.908	0.4236	0.8646	0.5852	0.7824
	*Forage type effect*				
Alfalfa	43.2b	15.9	35.1a	25.5a	130.1a
Cassava	47.9a	13.5	30.0b	21.2b	119.3b
*P*-value	0.0005	0.1641	< 0.0001	0.0003	0.0053
	*Banana flour addition effect*				
No banana	42.6a	14.0	33.7a	21.5b	118.9b
Banana	48.6b	15.4	31.4b	25.2a	130.5a
*P*-value	< 0.0001	0.4022	0.023	0.0012	0.0028
SEM	3.26	4.77	2.60	2.90	9.90

SEM: standard error of the mean, values for different comparisons in the same column with different letters are significantly different (*P* < 0.05). T_1_: alfalfa: grass hay at ratio of 30: 70, T_2_: alfalfa: grass hay: banana flour 30:60:10, T_3_ and T_4_ diets prepared by replacing alfalfa in T_1_ and T_2_ with cassava foliage, respectively.

#### Ruminal pH, ammonia concentration, nutrient degradability, and protozoal count

Results of the comparison between alfalfa diets vs. cassava diets for ruminal pH, NH_3_-N concentration, DOM, DNDF and protozoal count are presented in Table 5. The values of pH after 48 h of incubation did not differ significantly among tested diets, also there was no significant effect of forage (alfalfa vs. cassava). However, banana flour addition to cassava or alfalfa diets showed a significant (*P* < 0.05) reduction in pH values compared with diets without banana flour being 5.92 and 6.0, respectively. Concerning NH_3_-N results, the values for NH_3_-N concentration ranged from 36.2 to 41.2 mg/100 ml. There were insignificant (*P* > 0.05) differences among the experimental diets. Also, neither forage type nor banana flour addition have a significant effect. Regarding DOM results, there were insignificant (*P* > 0.05) differences among the experimental diets, however the alfalfa diets (T_1_, T_2_) showed a significant (*P* < 0.05) higher DOM value vs. cassava diets (T_3_, T_4_) being 704.3 and 631.4 g/kg, respectively. The DNDF results indicated that there was a significant (*P* < 0.05) difference among the experimental diets. The DNDF highest value was recorded for T_1_ being 471.3 g/kg, while T_4_ recorded the lowest value being 203.3 g/kg. The addition of banana flour to diets significantly (*P* < 0.05) decreased the DNDF, being 328.0 g/kg vs. 419.3 g/kg for diets without banana flour. There was no-significant difference between tested groups in the main effects or the interaction between them for protozoal count. The values were ranging from 5.63 × 10^5^/ml for T_4_ and 6.90 × 10^5^/ml for T_1_.

**Table 5:** In vitro ruminal pH, ammonia (NH_3_-N) concentrations, degraded organic matter (DOM), degraded neutral detergent fiber (DNDF), and protozoal count for alfalfa versus cassava diets.

Diets	pH	NH3-*N* (mg/100 ml)	Degradability (g/kg)		Protozoal count (10^5^/ml)
			DOM	DNDF	
T_1_	5.96	37.2	693.5	471.3a	6.90
T_2_	5.90	39.3	715.0	452.7ab	6.15
T_3_	6.04	36.2	629.4	367.3b	5.85
T_4_	5.93	41.2	633.5	203.3c	5.63
*P*-value	0.2824	0.7003	0.5741	0.0268	0.7401
	*Forage type effect*				
Alfalfa	5.93	38.3	704.3a	462.0a	6.53
Cassava	5.98	38.7	631.4b	285.3b	5.74
*P*-value	0.0503	0.9076	0.0004	< 0.0001	0.3285
	*Banana flour addition effect*				
No banana	6.00a	36.7	661.4	419.3a	6.38
Banana	5.92b	40.3	674.3	328.0b	5.89
*P*-value	0.008	0.3552	0.4086	0.0081	0.5401
SEM	0.05	6.75	29.99	57.65	1.55

SEM: standard error of the mean, values for different comparisons in the same column with different letters are significantly different (*P* < 0.05). T_1_: alfalfa: grass hay at ratio of 30: 70, T_2_: alfalfa: grass hay: banana flour 30:60:10, T_3_ and T_4_ diets prepared by replacing alfalfa in T_1_ and T_2_ with cassava foliage, respectively.

Table 6 shows the comparison between alfalfa and cassava diets for individual and total SCFA’s. There were no significant differences among the experimental diets for the individual SCFA’s except for valerate. The highest valerate value was recorded for T_1_ being 1.83%, while T_3_ and T_4_ recorded the lowest values being 1.51 and 1.47%, respectively. In the comparison of alfalfa and cassava diets, the proportions of the individual SCFA’s (except for acetic) significantly differed between alfalfa and cassava diets to be higher for isobutyrate, isovalerate and valerate in alfalfa diets. The production of acetate in diets with banana was significantly (*P* = 0.0018) lower than the other diets without banana being 65.9 and 66.9% of total SCFA’s, respectively.

**Table 6:** Molar proportions of individual and total short-chain fatty acids (SCFA’s) concentration for alfalfa versus cassava diets.

Diets	Molar proportions of individual SCFA’s (% of total SCFA’s)							
	Acetate	Propionate	Isobutyrate	Butyrate	Isovalerate	Valerate	Total SCFA’s mM	C_2_/C_3_ ratio
T_1_	66.6	15.4	2.87	10.6	2.68	1.83a	76.4	4.32a
T_2_	66.0	15.3	2.71	11.8	2.48	1.76b	80.8	4.31a
T_3_	67.2	15.5	2.63	11.7	1.51	1.51c	74.2	4.33a
T_4_	65.8	16.1	2.53	12.8	1.26	1.47c	76.0	4.08b
*P*-value	0.1329	0.0158	0.4755	0.7245	0.8390	0.1990	0.2702	0.0135
	*Forage type effect*							
Alfalfa	66.3	15.4b	2.8a	11.2b	2.58a	1.79a	78.6a	4.31a
Cassava	66.5	15.8a	2.6b	12.3a	1.39b	1.50b	75.1b	4.20b
*P*-value	0.3883	0.0047	0.0009	< 0.0001	< 0.0001	< 0.0001	0.0133	0.0202
	*Banana flour addition effect*							
No-banana	66.9a	15.5	2.8a	11.1b	2.10	1.67a	75.3b	4.33a
Banana	65.9b	15.7	2.6b	12.3a	1.87	1.62b	78.4a	4.19b
*P*-value	0.0018	0.0573	0.0134	< 0.0001	0.0956	0.0021	0.0230	0.0080
SEM	0.38	0.21	0.07	0.16	0.21	0.02	1.93	0.07

C_2_/C_3_: acetate to propionate ratio. SEM: standard error of the mean, values for different comparisons in the same column with different letters are significantly different (*P* < 0.05). T_1_: alfalfa: grass hay at ratio of 30: 70, T_2_: alfalfa: grass hay: banana flour 30:60:10, T_3_ and T_4_ diets prepared by replacing alfalfa in AG and AGB with cassava foliage, respectively.

A significant difference was observed in the acetate to propionate ratio (C_2_/C_3_) between the tested diets. As a main effect, the cassava diets exhibited a lower C_2_/C_3_ ratio compared to the alfalfa diets, being 4.20 and 4.31, respectively. Additionally, the addition of rejected banana decreased the C_2_/C_3_ ratio to 4.19, compared to 4.33 for diets without banana addition.

## Discussion

### The most abundant chemical components of cassava foliage

Isoginkgetin was the major component among the identified compounds in cassava foliage extract, with a peak area percentage of 25.33%. Isoginkgetin has been associated with various biological activities, including antitumor and anti-inflammatory effects^33^. Another powerful anti-inflammatory component detected in cassava foliage is Scutellarein tetramethyl ether. The potential uses and mode of action of Scutellarein tetramethyl ether as a treatment of inflammatory-related diseases have been demonstrated by^34^. Scutellarein tetramethyl ether acts as a polymerase chain inhibitor, specifically targeting the Cyclooxygenase-2 enzyme implicated in inflammation and pain. It exhibits apoptosis pathway inhibition, possibly due to its ability to inhibit protein synthesis by binding to the ribosome^35^. Additionally, this compound also targets Toll-like receptor 4, aiding in the inflammatory response to bacterial infection^36^. Scutellarein tetramethyl ether has also demonstrated anti-cancer properties in vitro^34^. Cassava foliage is a rich source of vitamins^37^. Results of GC-MS analysis in the present study indicated the presence of retinal, vitamin E, and β carotene in cassava foliage in adequate amounts.

### Phenolic compounds of cassava foliage

Among the determined phenolic components, rutin (phenolic glycoside) recorded the highest value in the HPLC quantitative analysis of the ethanolic extract for cassava foliage. Rutin contains only a single flavonoid unit and is not a true tannin^38^. Rumen microbes may be capable of degrading rutin to produce energy. Rutin fermentation, with no other fermentable substrate, resulted in the production of 2.5 mol of acetate^39^.

Cassava foliage showed a high content of gallic acid and ferulic acid, phenolic components, both of which displayed a wide range of biological activities. Gallic acid, classified as gallotannins, is responsible for the formation of cassava foliage tannins content by esterifying partially or wholly the tannins’ central core (polyhydric alcohol)^40^. In the ethanolic extract, gallic acid was identified the principal phenolic acid with the highest value (144.25 µg/ml extract). This finding aligns with the observations of Laya and Koubala^11^, who reported a high abundance of gallic acid in cassava leaves. Gallic acid is known to offer health benefits, such as antioxidant and potential hepatoprotective effects^41^. Additionally, beneficial effects have been reported for gallic acid, and catechin (class: flavanols), including antioxidant, anti-inflammatory, and pharmacological properties^42–45^. On the other hand, chlorogenic acid has been found to have hypoglycemic, hypolipidemic, anti-inflammatory, antioxidant, and other pharmacological properties^46^.

### In vitro gas production and fermentation kinetics of tested diets

The current results revealed that cassava diets, constituting 30% of diet DM, reduced accumulative GP (after 24 h of incubation) by approximately 8.3% compared to alfalfa diets. While in the present study, diets containing either cassava or alfalfa have almost the same concentration of total tannin, cassava was found to have high condensed tannins^47^. The negative relationship between GP and inclusion of some tanniferous plants in the ruminants’ diets has been observed by several authors^48–50^. The reduction in GP may result from the antimicrobial properties of tannins and other phenolic compounds in these plants^51^. It could be also be attributed to the reduction in microbial enzymatic degradation and microbial growth due to the ability of condensed tannins to bind with fiber and protein^52–54^. Chaji et al.^55^ explained the reduction in GP by the disruptive effect of tannins on rumen microorganisms.

The in vitro incubation of feeds with buffered rumen fluid allows the fermentation of carbohydrates (structural and nonstructural) to produce SCFA’s, gases, and microbial cells. The difference in GP values reflects the consequences of carbohydrate fermentation (GP from protein and fat fermentation is relatively small versus carbohydrates) to acetate and butyrate. The lower gas production is associated with propionate production, which results from the buffering of the acid^13^. Therefore, the addition of banana flour increased the carbohydrate content in the diets, influencing the values of accumulative GP during the incubation time.

^39,56^ reported that the rapid degradation of alfalfa protein by ruminants can reduce the nitrogen utilization, leading to an increase in the cost of protein supplementation and increase nitrogen excretion, contributing to environmental pollution. Therefore, inclusion of rich tannin forages in ruminant diets, such as cassava foliage, could increase nitrogen utilization by binding tannins with protein, decreasing rumen protein degradability^57^, and enhancing absorption from lower gut^58^.

Results of protozoal count indicated that phenolic structures may disrupt protozoal membranes, inactivate protozoal enzymes, and deprive protozoa of substrates and metal ions essential for cell metabolism^59^. Consequently, this could be interpreted as a decrease in GP and CH_4_ emission. The current results indicated no significant difference in protozoal count among the tested diets, aligning with the findings of Wallace et al.^60^ and Saminathan et al.^61^, who mentioned that reducing digestibility with condensed tannins diets without affecting rumen microorganisms may be attributed to the inhibitory effect of condensed tannins on enzymatic activity or ruminal bacteria. The diversity response of rumen degradability to the inclusion of tannin-rich plants may be related to differences in molecular weight and chemical structure of condensed tannins, which are affected by factors such as species, genotype and growth stage of these plants^62,63^.

Cassava diets were higher in propionate and butyrate compared with alfalfa diets. Similar results for propionate and butyrate were mentioned by^64^ when goats were fed cassava foliage up to 75% of total dry matter intake. This could be a reason of higher N retention with diets supplemented with cassava foliage or due to a better balance between N and energy yielding substrates for ruminal micro-organism, leading to an increase in the capture of degradable N, microbial growth rate, and efficiency^65^. Regarding the banana addition effect on the fermentable carbohydrates, the current study demonstrated more propionate and lower acetate to propionate ratio for rejected banana diets. The slowly fermentable carbohydrates yield relatively higher acetate as compared to propionate^13^. However, it could be a result of low CP content of rejected banana diets.

Based on the bioactive compounds present in cassava foliage, further studies are necessary to determine the effects of phenolic and flavonoid compounds on the microbial communities of the rumen. Moreover, long-term in vivo studies are required with different species of ruminants, and feeding regimens to verify and rule out effects of cassava foliage on the health of animals, as well as the quality of meat and milk.

## Conclusion

The previous results illustrate that cassava foliage possess phenolic and flavonoids compounds; such as isoginkgetin, scutellarein tetramethyl ether, rutin, gallic acid and ferulic acid; which display a wide range of biological activities and they could have a positive effect on animal health and production. In addition, cassava foliage could be used as a suitable feed source in ruminant ration with the dietary supplementation with energy source like cull banana fruits to cover the nutritional requirements of animals.

## Data Availability

The datasets generated and/or analyzed during the current study are available from the corresponding author on reasonable request.
